# Supplementary material for: Empirical methods for controlling false positives and estimating confidence in ChIP-Seq peaks
Source: BMC Bioinformatics. 2008 Dec 5;9:523. doi: 10.1186/1471-2105-9-523 (PMC2628906; doi:10.1186/1471-2105-9-523)
Supplement: Additional File 1 — A variety of html documents from the USeq web site detailing the available applications, their best usage, output file type descriptions, command line menus, etc. [file 1471-2105-9-523-S1.zip › USeqUserGuides/JavaDoc.zip!/JavaDoc/trans/roc/class-use/Bin.html]

Uses of Class trans.roc.Bin


|  |  |  |
| --- | --- | --- |
| **Overview** \| **Package** \| **Class** \| **Use** \| **Tree** \| **Deprecated** \| **Index** \| **Help** | |  |
| PREV   NEXT | **FRAMES**    **NO FRAMES**     **All Classes** |  |


---


## **Uses of Class trans.roc.Bin**

| Packages that use Bin | |
| --- | --- |
| **trans.roc** | For manipulating the BAC dmel spike in data and generating ROC curves. |

Uses of Bin in trans.roc

| Methods in trans.roc that return Bin | |
| --- | --- |
| `Bin` | `SgrBacCounter.findBin(Sgr sgr)`             Finds an appropriate bin given an sgr object |
| `Bin` | `MethodTester.findBin(Sgr sgr)`             Finds an appropriate bin given an sgr object |

---


|  |  |  |
| --- | --- | --- |
| **Overview** \| **Package** \| **Class** \| **Use** \| **Tree** \| **Deprecated** \| **Index** \| **Help** | |  |
| PREV   NEXT | **FRAMES**    **NO FRAMES**     **All Classes** |  |


---
